# Retroperitoneal Schwannoma: Two Rare Case Reports

**DOI:** 10.7759/cureus.13456

**Published:** 2021-02-20

**Authors:** Marouane Harhar, Abdelbassir Ramdani, Tariq Bouhout, Badr Serji, Tijani El Harroudi

**Affiliations:** 1 Surgical Oncology, Mohammed VI University Hospital, Regional Oncology Center, Oujda, MAR

**Keywords:** schwannoma, retroperitoneal tumor

## Abstract

Schwannomas are neuroectodermal tumors that rarely occur in the retroperitoneal space. We report two cases of patients who presented with abdominal pain. Radiological findings revealed a retroperitoneal mass in both cases. Both patients underwent complete surgical excision with an uneventful postoperative course. The histopathological study confirmed the nature of schwannoma. Complete surgical excision remains the gold standard for the management of these tumors. The preoperative diagnosis is usually difficult; however, the definitive diagnosis is made upon histopathological examination.

## Introduction

Schwannomas are neuroectodermal tumors that usually affect nerves in the head, neck, and extremities. The retroperitoneal location is rare and accounts for approximately 3% of all schwannoma [1]. Retroperitoneal schwannomas (RSS) represent 4% of all retroperitoneal tumors [2]. They are essentially benign tumors, however, malignant forms have been reported in up to 60% of patients with Von Recklinghausen’s disease [3]. Generally, these tumors are clinically silent and discovered incidentally, and the symptoms are vague and nonspecific [4]. Sometimes, the preoperative diagnosis is hard to define. Histologically, schwannomas are typically characterized by the presence of areas of high cellularity (Antoni A tissue), and low cellularity (Antoni B tissue) [5]. The therapeutic approach depends on the pre-operative evaluation, the size and location of the mass, and the surgeon’s preference [6]. Herein we present the clinical, biological, and radiological features of two patients with schwannoma and describe the therapeutic management conducted in both cases. Written consent was obtained by all participants in this study.

## Case presentation

Case 1

**Figure 1:**
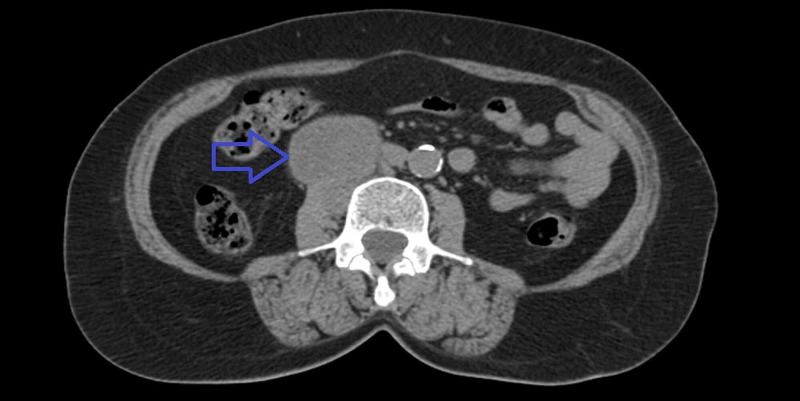
Axial abdominal CT scan showing a right retroperitoneal mass (blue arrow).

**Figure 2:**
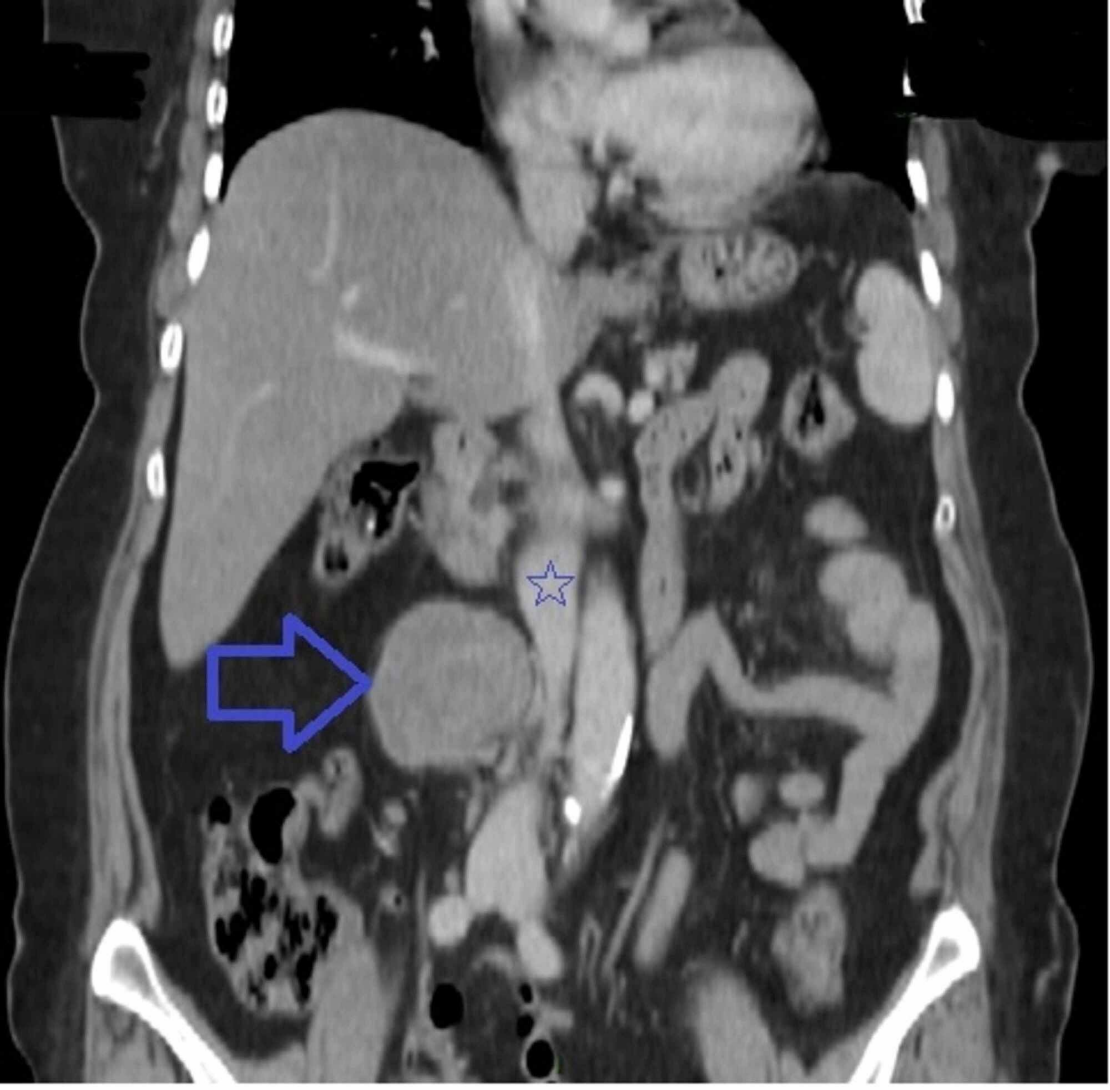
Coronal abdominal CT scan showing a retroperitoneal mass (blue arrow) located in the lateral and retrocaval area with intimate contact with the inferior vena cava (blue star).

A 70-year-old female patient, with a history of diabetes, presented with chronic right flank abdominal pain. Abdominal ultrasound and computed tomography scan (CT) revealed a heterogenous right retroperitoneal mass, localized in the lateral and retrocaval area and medial to the right kidney, measuring 10 x 5 cm (Figures 1, 2).

**Figure 3:**
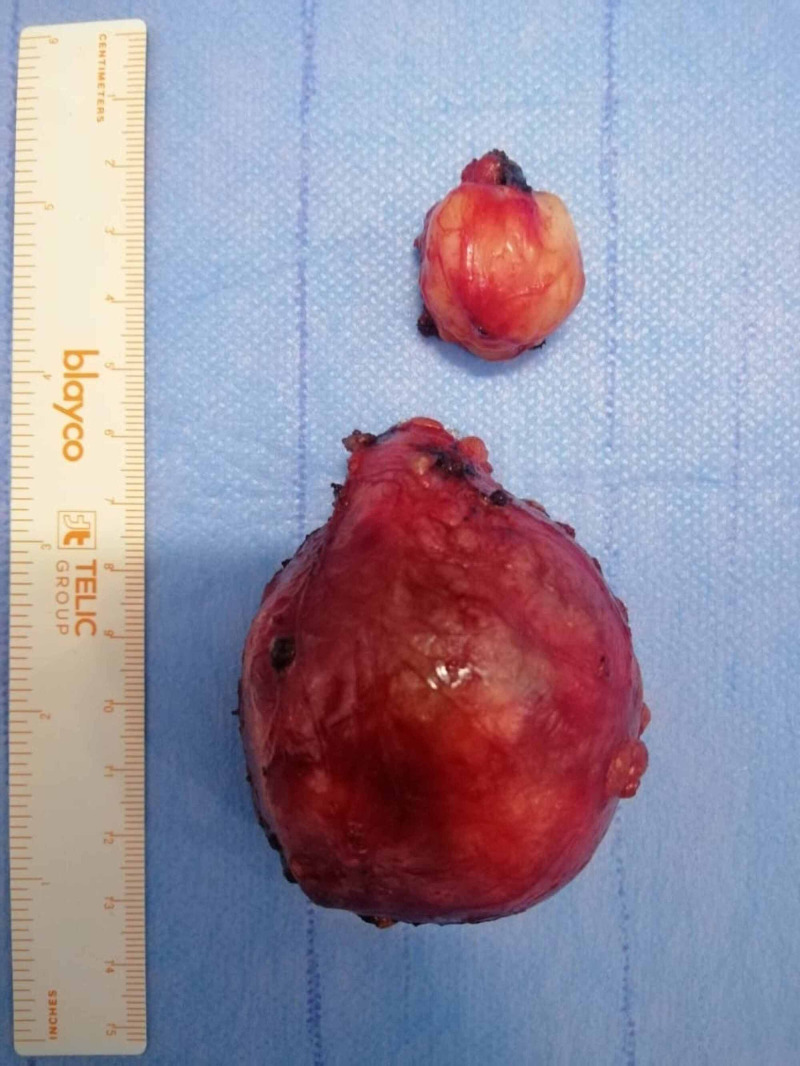
Image showing the resected mass.

Laboratory tests including tumor markers and urine catecholamine were normal. The patient underwent a laparotomy surgery, showing a bilobed mass connected to the intervertebral foramina, and making us suspect the possibility of schwannoma. Thus, a complete resection of the tumor was performed (Figure 3).

The postoperative course was uneventful. Microscopic findings revealed the presence of spindle-shaped cells with a typical palisading pattern, arranged in short bundles of interlacing fascicles. The final pathologic diagnosis was a benign schwannoma. The patient experienced no recurrence over six months of follow-up

Case 2

**Figure 4:**
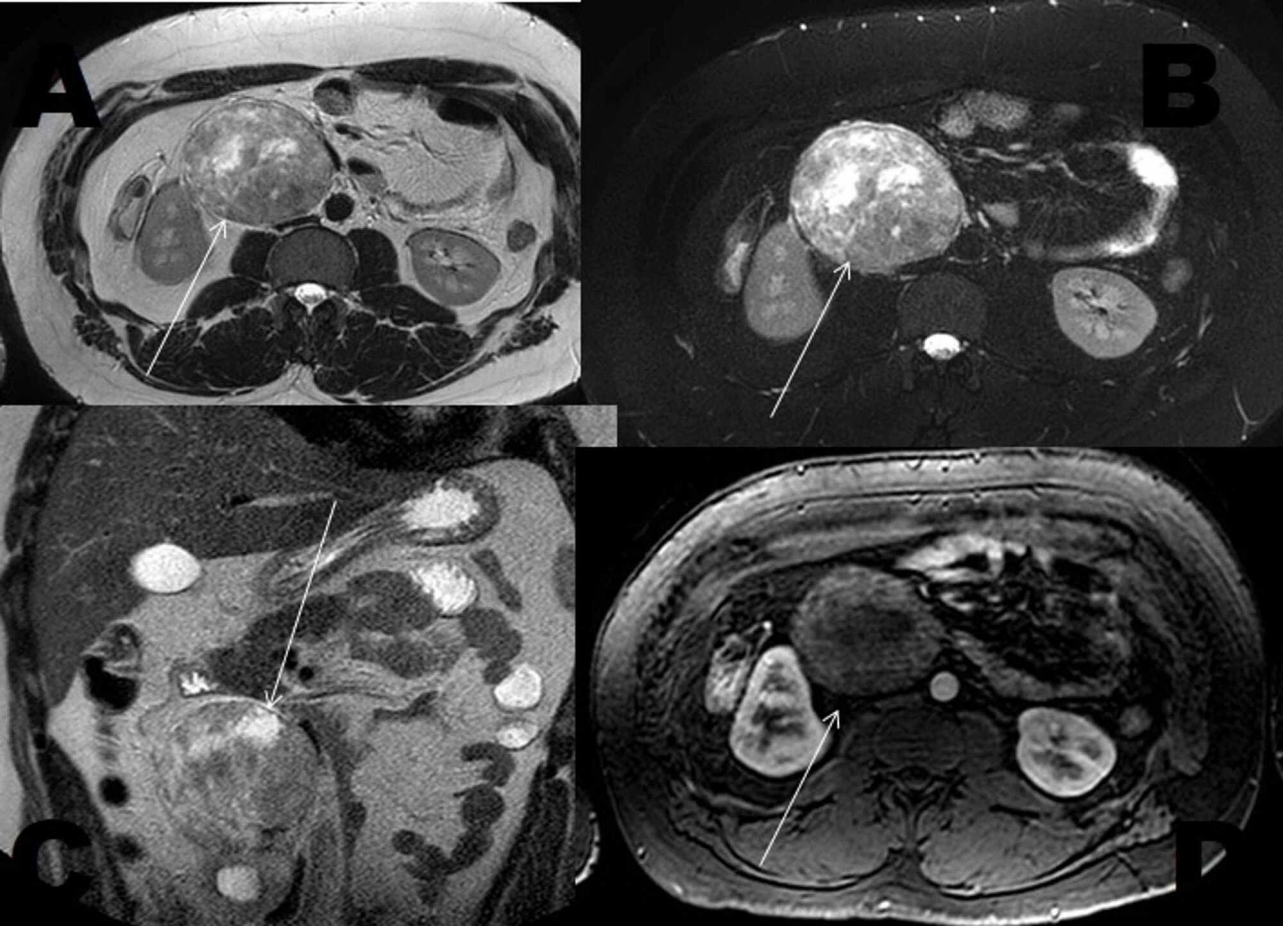
Abdominal MRI: Axial sections in T2 sequence (A), T2 with fat saturation (B), T2 coronal sections (C), and axial T1 sections with fat saturation and gadolinium contrast (D) showing a large retroperitoneal mass (arrows), well-circumscribed, hyperintense in T2 sequence, heterogeneous with cystic areas not enhanced by contrast.

A 29-year-old male patient, with no noticeable medical or surgical history, presented with a one-year history of chronic diffuse abdominal pain, physical examination was unremarkable. Abdominopelvic CT scan and abdominal MRI showed a well-circumscribed, heterogenous retroperitoneal mass developed in the right lateral aortic area measuring 14 x 7 cm (Figure 4).

The patient underwent an exploratory laparotomy. Intraoperatively, the mass was located in the lateral aortic area and connected to the intervertebral foramina. We decided to perform a complete resection of the mass.

Pathological examinations revealed a benign schwannoma. The patient made an uneventful recovery, he remained asymptomatic, with no recurrence at a one-year follow-up.

## Discussion

Schwannomas are neurogenic tumors deriving from the Schwann cells of nerve sheaths, affecting predominantly females and mostly found in patients aged 20 to 60 years [7]. The retroperitoneal location accounts for approximately 3% of all schwannoma. Schwannomas are usually benign tumors, and scarcely undergo malignant transformation unless they are associated with von Recklinghausen's disease that accounts for 5% to 18% of all RSs [1].

Considering the flexible feature of the retroperitoneum, the RSs are usually clinically “silent” and need to reach a significant size to be responsible for compressive symptoms, ranging from vague abdominal pain to abdominal mass and urinary difficulties. In our cases, abdominal pain was the predominant symptom.

Radiologic examination plays a major role in the diagnostic approach. CT scan with contrast shows classically a well-defined tumor with slightly low density compared with soft tissue due to the high presence of myelin and fat [8]. MRI is superior to CT in defining the margins and identifying the nerve origin [9]. Schwannomas are commonly hypointense on T1 and hyperintense on T2-weighted MR images [8]. The target sign (a hypointense center with a hyperintense periphery) and fascicular sign (bundles) are two well-known characteristic features of neurogenic tumors on MRI; however, they are not seen frequently in RSs [10]. Malignant schwannomas have irregular contours, exhibit mixed intensity on both T1- and T2-weighted images, and tend to invade other structures; however, they are extremely rare in patients without a history of neurofibromatosis [9,11].

The differential diagnoses with schwannomas include neurogenic tumors such as paraganglioma and pheochromocytoma; tumors such as fibrosarcoma, liposarcoma, and ganglioneuroma have similar findings on CT and MRI scans making preoperative diagnosis unlikely [11,12].

Percutaneous biopsy is a matter of debate. Some physicians skipped it due to the possibility of biopsy-related complications (bleeding, infection, tumor seeding) while other physicians managed patients conservatively due to benign biopsy results [6,13,14]. In our two cases, we took into consideration the possible complications of biopsy as well as the existence of symptoms related to the size of the tumor that needed to be excised to improve the symptoms.

The final diagnosis is solely based on histopathologic examination of the specimens. RS is histologically characterized by alternating Antoni A and Antoni B areas [5]. In Antoni A tissue, the cells are arranged in an organized compact pattern, while in Antoni B tissue, the cells are scattered loosely in an edematous matrix [5,13,15]. Immunohistology complement shows strong and diffuse staining of S-100 protein in schwannomas cells cytoplasm which confirms the diagnosis of schwannoma [11].

Surgical management consists of total resection. These tumors are usually solitary, well-circumscribed, and noninvasive, so complete surgical excision provides good results [16]. The surgical difficulties are essentially related to the risk of injuring surrounding neurovascular structures and devascularization of the ureter during tumor dissection. In our case, the surgical exploration found a connection between the tumor and the intervertebral foramen suggesting the diagnosis of schwannoma intraoperatively. The laparoscopic approach is feasible and only a few cases are reported in the English literature [7]. It exhibits a promising future in the treatment but the choice of surgical management may largely rely on the size and location of tumors [7].

Recurrence of benign schwannoma is rare, it follows usually incomplete resection, which was reported in 5%-10% of cases [17]. In case of recurrence, surgical excision is recommended. Adjuvant therapy is not recommended due to the lack of sensitivity of this tumor to chemotherapy and radiotherapy [18].

## Conclusions

Retroperitoneal schwannomas are rare tumors. They are mostly benign, but can rarely be malignant, especially when associated with neurofibromatosis. The final diagnosis is based on histological and immunohistochemical findings of the specimens. Complete surgical resection remains the gold standard for the management of RSs. Despite the rare location of schwannoma in the retroperitoneum, they should be considered in cases of retroperitoneal abdominal masses.

## References

[1] Daneshmand S, Youssefzadeh D, Chamie K (2003). Benign retroperitoneal schwannoma: a case series and review of the literature. Urology.

[2] Felix EL, Wood DK, Das Gupta TK (1981). Tumors of the retroperitoneum. Curr Prob Cancer.

[3] Sengar Hajari AR, Tilve AG, Kulkarni JN, Bharat R (2015). Malignant peripheral nerve sheath tumor of the uterine corpus presenting as a huge abdominal neoplasm. J Can Res Ther.

[4] Goh BK, Tan YM, Chung YF, Chow PK, Ooi LL, Wong WK (2006). Retroperitoneal schwannoma. Am J Surg.

[5] Pilavaki M, Chourmouzi D, Kiziridou A, Skordalaki A, Zarampouzas T, Dervelengas A (2004). Imaging of peripheral nerve sheath tumors with pathologic correlation: pictorial review. Eur J Radiol.

[6] Chiang HC, Yan MY, Chen CA, Chen PH (2018). Giant retroperitoneal schwannoma: a case treated with 3D laparoscopic resection. Biomed Sci & Tech Res.

[7] Rajkumar JS, Ganesh D, Anirudh JR, Akbar S, Kishore CM (2015). Laparoscopic excision of retroperitoneal schwannoma. J Clin Diagn Res.

[8] Dublin AB, Dedo HH, Bridger WH (1995). Intranasal computed schwannoma: magnetic resonance and computed tomography appearance. Am J Otolaryngol.

[9] Fernandez RB, Villamil LR, Montes SF (2016). Management of retroperitoneal schwannoma reports and review of the literature. World J Nephrol Urol.

[10] Hughes MJ, Thomas JM, Fisher C, Moskovic EC (2005). Imaging features of retroperitoneal and pelvic schwannomas. Clin Radiol.

[11] Zhang L, Gao M, Zhang T, Chong T, Wang Z, Liu W, Li H (2018). Surgical management of retroperitoneal schwannoma complicated with severe hydronephrosis: a case report. Medicine.

[12] Kalaycı M, Akyüz U, Demirağ A, Gürses B, Ozkan F, Gökçe O (2011). Retroperitoneal schwannoma: a rare case. Case Rep Gastrointest Med.

[13] Al Skaini MS, Haroon H, Sardar A (2015). Giant retroperitoneal ancient schwannoma: is preoperative biopsy always mandatory?. Int J Surg Case Rep.

[14] Strauss DC, Qureshi YA, Hayes AJ, Thomas JM (2011). Management of benign retroperitoneal schwannomas: a single-center experience. Am J Surg.

[15] Walter JB, Talbot IC (1965). General pathology. http://hdl.handle.net/123456789/2456.

[16] Singh M, Kumar L, Chejara R, Prasad OP, Kolhe Y, Saxena A (2014). Diagnostic dilemma of a rare, giant retroperitoneal schwannoma: a case report and review of literature. Case Rep Oncol Med.

[17] Gu L, Liu W, Xu Q, Wu ZY (2008). Retroperitoneal schwannoma mimicking hepatic tumor. Chin Med J.

[18] Fujimoto N, Kubo T, Hisaoka M (2018). Demographics, management and treatment outcomes of benign and malignant retroperitoneal tumors in Japan. Int J Urol.

